# Exploring the Association between Serum BDNF and Attempted Suicide

**DOI:** 10.1038/srep25229

**Published:** 2016-04-28

**Authors:** Rebecca B. Eisen, Stefan Perera, Monica Bawor, Brittany B. Dennis, Wala El-Sheikh, Jane DeJesus, Sumathy Rangarajan, Judith Vair, Heather Sholer, Nicole Hutchinson, Elizabeth Iordan, Pam Mackie, Shofiqul Islam, Mahshid Dehghan, Jennifer Brasch, Rebecca Anglin, Luciano Minuzzi, Lehana Thabane, Zainab Samaan

**Affiliations:** 1 MiNDS Neuroscience Graduate Program, McMaster University, 1280 Main Street West, Hamilton, ON L8S 4L8, Canada; 2 Health Research Methodology Graduate Program, McMaster University, 1280 Main Street West, Hamilton, ON L8S 4L8, Canada; 3 Department of Clinical Epidemiology and Biostatistics, McMaster University, 1280 Main Street West, Hamilton, ON L8S 4L8, Canada; 4 St. George’s University of London, London, Cranmer Terrace, London SW17 0RE, United Kingdom; 5 Population Health Research Institute, Hamilton General Hospital, 237 Barton Street East, Hamilton, ON L8L 2X2, Canada; 6 St. Joseph’s Healthcare Hamilton, 100 West 5th Street, Hamilton, ON L8N 3K7, Canada; 7 Biostatistics Unit, Centre for Evaluation of Medicine, 25 Main Street West Suite 2000, Hamilton, ON L8P 1H1, Canada; 8 Department of Psychiatry and Behavioural Neurosciences, McMaster University, 1280 Main Street West, Hamilton, ON L8S 4L8, Canada; 9 Department of Medicine, McMaster University, 1280 Main Street West, Hamilton, ON L8S 4L8, Canada; 10 Women’s Health Concerns Clinic, St. Joseph’s Healthcare Hamilton, 50 Charlton Avenue East, Hamilton, ON L8N 4A6, Canada; 11 System-Linked Research Unit on Health and Social Service Utilization, McMaster University, 1280 Main Street West, Hamilton, ON L8S 4L8, Canada; 12 Peter Boris Centre for Addiction Research, St. Joseph’s Healthcare Hamilton, 100 West 5th Street, Hamilton, ON L8P 3R2, Canada

## Abstract

Suicide is a leading cause of death and a significant public health concern. Brain-derived neurotrophic factor (BDNF), a protein important to nervous system function, has been implicated in psychiatric disorders and suicidal behaviour. We investigated the association between serum levels of BDNF and attempted suicide in a sample of 281 participants using a case-control study design. Participants were recruited from clinical and community settings between March 2011 and November 2014. Cases (individuals who had attempted suicide) (n = 84) were matched on sex and age (within five years) to both psychiatric controls (n = 104) and community controls (n = 93) with no history of suicide attempts. We collected fasting blood samples, socio-demographic information, physical measurements, and detailed descriptions of suicide attempts. We used linear regression analysis to determine the association between BDNF level (dependent variable) and attempted suicide (key exposure variable), adjusting for age, sex, body mass index, current smoking status, and antidepressant use. 250 participants were included in this analysis. In the linear regression model, attempted suicide was not significantly associated with BDNF level (β = 0.28, SE = 1.20, P = 0.82). Our findings suggest that no significant association exists between attempted suicide and BDNF level. However, the findings need to be replicated in a larger cohort study.

Suicide claims nearly one million lives each year, making it a leading cause of death worldwide and a significant public health concern^1^. The devastating effects of suicide are felt at the family, community, and societal levels. Suicidal thoughts, plans, and acts intended to end one’s life all comprise the complex phenomenon of suicidal behaviour. Non-fatal suicidal behaviours are 10–20 times more common than completed suicide^2^. Attempted suicide is also an important risk factor for future completed suicide^1^.

Many risk factors are thought to contribute to the risk of suicidal behaviour. These include biological, psychological, social, and environmental factors^3^,^4^,^5^. Psychiatric disorders are highly predictive of suicidal behaviour, among which mood disorders pose significant risk^5^. Population level estimates suggest that 90% of attempted and completed suicides occur in the context of a psychiatric disorder^3^. Other known risk factors include chronic illness, substance use disorders, and demographic variables such as age and sex^3^. These biological and psychological factors point to a predisposition toward suicidal behaviour in some individuals. However, these factors alone do not predict suicidal behaviour. Social and environmental risk factors also play a role, and include unemployment, low educational attainment, unmarried status, and a lack of social support^3^. Incidents of suicidal behaviour likely result from the interaction between biological and psychosocial factors.

Brain-derived neurotrophic factor (BDNF) is the most abundant member of the neurotrophins, a family of proteins that regulate the survival, development, maintenance, and function of vertebral nervous systems^6^. BDNF is involved in many neural processes including neurogenesis, nerve growth, neuroplasticity, and neurotransmission^7^. Altered levels of BDNF have been associated with several psychiatric conditions.

Low blood levels of BDNF have been linked to depression^8^,^9^ and reduced BDNF expression in the brain has been linked to stress^10^,^11^. Both depression and stress are major risk factors for suicidal behaviour^7^. Since BDNF is intrinsic to optimal nervous system function, pathological changes in BDNF levels are a possible cause of neurobiological deficits that impair one’s ability to adapt to difficult situations^7^.

Recent research has examined the association between BDNF and suicidal behaviour^12^,^13^,^14^,^15^,^16^,^17^,^18^,^19^,^20^,^21^,^22^,^23^. The literature on this topic has been summarized and evaluated in a systematic review and meta-analysis by Eisen *et al*.^24^. Some studies have investigated postmortem levels of BDNF in the brains of suicide victims^12^,^13^,^14^, while other studies have measured peripheral BDNF levels in clinical samples^15^,^16^,^17^,^18^,^19^,^20^,^21^,^22^,^23^. Postmortem studies have found significantly lower BDNF levels in the hippocampus and prefrontal cortex in individuals who died by suicide compared to individuals who died of other causes^12^,^13^,^14^. Studies comparing peripheral levels of BDNF in individuals with and without a history of suicidal behaviour have shown conflicting results^15^,^16^,^17^,^18^,^19^,^20^. Some studies of serum levels of BDNF have shown significantly reduced levels in individuals with suicide attempts compared to both psychiatric and healthy controls^15^,^16^. However, other studies making the same comparison found no significant relationship^17^,^18^,^19^,^20^. Studies of plasma BDNF levels in individuals with depression with and without a history of suicidal behaviour are similarly conflicted in their findings^21^,^22^,^23^.

Studies of the association between BDNF levels and suicidal behaviour are few in number and limited in methodology. The sample sizes are generally small, with some comparison groups containing as few as 10 participants (for examples, see^17^,^18^). As well, nearly all previous studies conducted univariate analyses to compare BDNF levels among patient groups, and few studies adjusted for confounding variables in their analyses. Furthermore, many previous studies compare BDNF levels between groups of individuals with and without a lifetime history of attempted suicide (for examples, see^15^,^18^,^22^). In these studies, the BDNF measurements may not represent BDNF levels at the time of the attempt. Additional research is required to establish the relationship between BDNF levels and suicidal behaviour.

In the present study we examine the relationship between serum BDNF levels and recent suicide attempts (within the past three months) in a large clinical sample using a case-control study design.

## Results

**Figure 1:**
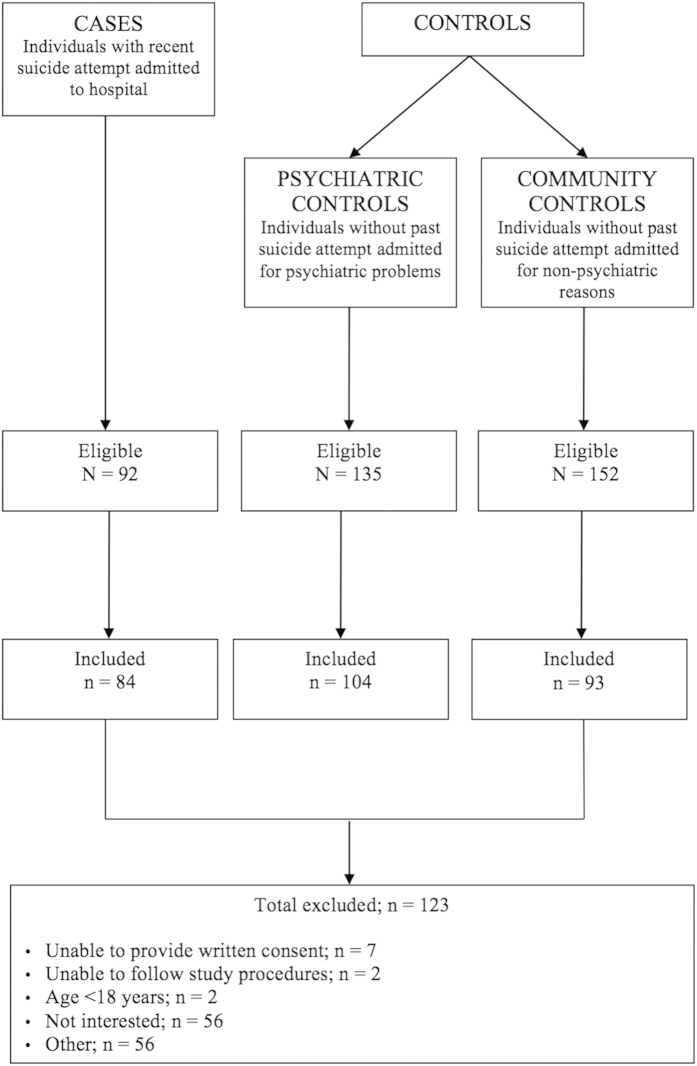
Flow diagram for participants included in the DISCOVER Study.

**Figure 2:**
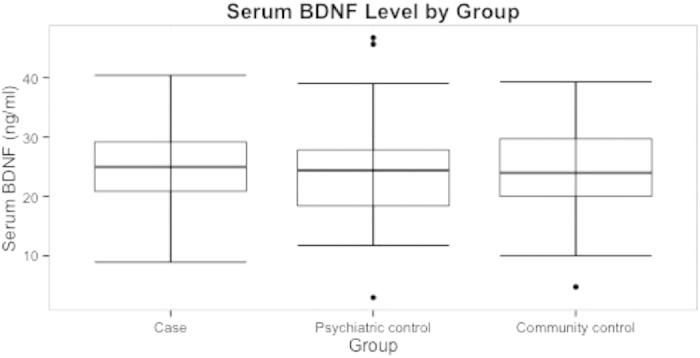
Serum BDNF Level by Group. The mean serum BDNF level was 24.21 (7.19) ng/ml in the case group, 23.88 (6.95) ng/ml in the psychiatric control group, and 24.77 (7.19) ng/ml in the community control group. No significant differences were found among groups (P = 0.59).

A total of 281 participants were recruited, including 84 cases (individuals who had attempted suicide), 104 psychiatric controls, and 93 community controls (see Fig. 1 for flow diagram of recruitment). The mean age of the sample was 43.7 years (standard deviation [SD] = 14.6, range: 18–73). The sample contained approximate equal numbers of males and female participants (52.2% female). The three groups differed significantly on a number of demographic variables including education level and employment status, with the case group containing the fewest individuals with secondary education and current employment. Several psychiatric disorders were most common in the case group, including major depressive disorder, anxiety disorders, alcohol and substance abuse, and antisocial personality disorder. The mean BDNF level was 24.21 ng/ml (SD = 7.19) in the case group, 23.88 ng/ml (SD = 6.95) in the psychiatric control group, and 24.77 ng/ml (SD = 7.01) in the community control group (see Fig. 2). The sample characteristics are summarized in Table 1.

Mean BDNF levels for the largest psychiatric disorder categories (mood disorders and anxiety disorders) were compared using ANOVA. In both categories, no significant differences were found in mean BDNF level among cases, psychiatric controls, and community controls (F = 0.55, P = 0.58 for mood disorder; F = 0.20, P = 0.82 for anxiety disorder).

### Primary Analysis

The linear regression analysis did not demonstrate a significant association between attempted suicide and BDNF level (β = 0.28, standard error [SE] = 1.20, P = 0.82). Of the covariates included (age, sex, smoking status, body mass index, and antidepressant use), only antidepressant use was significantly associated with BDNF level (β = −2.50, SE = 0.99, P = 0.012). The analysis included 250 out of 281 participants (11% missing data). The assumptions of linearity, independence, normality, and homoscedasticity were tested and all were satisfied. The variance inflation factors (VIF) for all variables were below 1, indicating that multicollinearity was not a concern. See Table 2 for linear regression model.

When this analysis was repeated with the inclusion of mood disorders and anxiety disorders as covariates, neither was significantly associated with BDNF level β = −0.79, P = 0.54 for mood disorder; β = 2.20, P = 0.07 for anxiety disorder). The overall results were the same as those of the primary analysis.

### Sensitivity Analysis

Linear regression analyses were conducted to examine the relationship between attempted suicide and BDNF level when cases are compared to each control group individually. In the first model cases were compared to psychiatric controls, and in the second model cases were compared to community controls. In both models, attempted suicide was not significantly associated with BDNF level (β = 0.85, SE = 1.13, P = 0.46 in the first model; β = 0.20, SE = 1.27, P = 0.87 in the second model).

## Discussion

Upon examining the association between serum BDNF level and attempted suicide using a case-control design, our findings demonstrate that serum BDNF level was not significantly associated with attempted suicide. The association was true regardless of comparison group.

Our study’s findings are not in accordance with the hypothesis that lower levels of BDNF are associated with suicidal behaviour. Our findings conflict with previous literature on this relationship, including a study by Dawood *et al*. that examined the association between internal jugular venous BDNF and suicidal behaviour^25^. Dawood *et al*. found a negative correlation between suicide risk and BDNF concentration, which supports the notion that reduced brain levels of BDNF are involved in the pathogenesis of depression and suicide. However, the study’s small sample size (16 participants) and unadjusted statistical analyses may have resulted in a biased estimate.

Only six previous studies have compared serum BDNF levels in groups with and without suicidal behaviour. While two of these studies found a significant association between serum BDNF and attempted suicide^15^,^16^, the remaining studies found no significant association^17^,^18^,^19^,^20^. While our study’s findings are not in accordance with the hypothesis that lower levels of BDNF are associated with depression and suicidal behaviour, they are consistent with other studies of this relationship.

This study has a number of strengths that distinguish it from other studies in this area. Our sample of 281 participants was larger than most previous studies, some of which included only 40 participants in total. As well, since our case group comprised individuals with recent suicide attempts (within three months of recruitment), the BDNF measurement was taken within a consistently short period of time relative to the attempt. Other studies have included participants with a lifetime history of suicide attempts. In these studies, the BDNF measurements may not represent BDNF levels near the time of the attempt. An additional strength of this study is the inclusion of two control groups, a psychiatric control group and a community control group. Since the majority of suicide attempts occur in the context of a psychiatric disorder^3^, it is important to include both comparisons in studies of suicidal behaviour. Finally, unlike most previous studies, we performed adjusted analyses to examine the relationship between BDNF level and attempted suicide. We adjusted for a number of variables known to be associated with altered BDNF level. The results of other studies may have been influenced by confounding variables such as age, smoking status, and body mass index.

Comparing BDNF levels among the three groups (cases, psychiatric controls, and community controls) within categories of psychiatric disorders (mood disorders and anxiety disorders) revealed that mean BDNF levels did not differ significantly within either the mood disorder or anxiety disorder categories. As well, when each of these diagnosis categories was include in the linear regression model, neither was significantly associated with BDNF level. While there is evidence to suggest that BDNF levels are related to several psychiatric disorders, including depression^8^,^9^, bipolar disorder^26^,^27^, schizophrenia^28^, and substance use disorder^29^,^30^, as of yet, it is unknown whether the relationship between BDNF and suicidal behaviour depends on the presence of an underlying psychiatric disorder. Future research should explore this topic.

Our finding that antidepressant use was associated with decreased BDNF level is contradictory to most previous evidence. In a meta-analysis conducted by Molendijk *et al*. in 2013, serum BDNF levels were found to be increased in antidepressant-treated patients with depression compared to untreated patients with depression^31^. The meta-analysis included 28 comparisons, of which only four found the opposite effect, with increased BDNF levels in untreated groups with depression^32^,^33^,^34^,^35^. These studies generally concluded that the effects of antidepressants on BDNF concentrations depends on the type of antidepressant used, and even within the same category of antidepressant (selective serotonin reuptake inhibitors, for instance), different antidepressants vary in their effects on BDNF level. Since all types of antidepressants were combined into one variable in our analysis, this could explain our unexpected result. Molendijk *et al*.’s meta-analysis also concluded that most studies of BDNF levels in antidepressant-treated and untreated individuals with depression are underpowered, and when publication bias is accounted for, the effect size is smaller than previously thought^31^. This puts into question the notion that high BDNF levels are in fact associated with antidepressant use. Another important point to consider is that of the 80 individuals in our sample who were taking antidepressants, 41% did not meet the requirements for major depressive disorder according to the Mini-International Neuropsychiatric Interview. These individuals could have been taking antidepressant medications for various reasons, including treatment of anxiety, chronic pain, or insomnia. This may partly explain our finding, since other factors may be responsible for the low BDNF levels in patients on antidepressants in our sample.

Future studies should aim to replicate these findings using a cohort study design. Additional research should also explore other potential biomarkers for suicidal behaviour. Since there is evidence for a connection between hyperactivity of the hypothalamic-pituitary-adrenal (HPA) axis and suicidal behaviour^2^, potential biomarkers could include cortisol or pro-inflammatory cytokines.

## Conclusion

Our case-control study shows no significant association between serum BDNF level and attempted suicide. However, the finding will need to be replicated in a larger cohort study.

## Methods

### Data Collection

The data used in this study were collected from the Study of Determinants of Suicide Conventional and Emergent Risk (DISCOVER), an observational matched case control study that aims to understand the risk factors involved in suicidal behaviour^36^. Cases (participants hospitalized for a suicide attempt) were matched on age and sex to two control groups (psychiatric patients and community controls). Data were collected at St. Joseph’s Healthcare Hamilton, Hamilton Health Sciences Hospitals, and the Hamilton City community, in Ontario, Canada. Data collection began in March 2011 and ended in November 2014. The study was approved by the Hamilton Integrated Research Ethics Board (HiREB) (number 10–661 for St. Joseph’s Healthcare Hamilton and 11–3479 for Hamilton Health Sciences Hospitals). The methods of this study were performed in accordance with the HiREB guidelines. This study follows the Strengthening the Reporting of Observational Studies in Epidemiology (STROBE) guidelines^37^.

### Inclusion and Exclusion Criteria

The study included men and women 18 years and older who were able to provide written informed consent, communicate in English, and follow study procedures. Cases were defined as individuals who made a serious suicide attempt (defined as self-directed injury with intent to die) within in the last three months, were admitted to hospital, and required medical or psychiatric intervention. Cases were matched based on sex and age (within five years) to both psychiatric and community control groups. The psychiatric control group consisted of individuals with psychiatric disorders with no history of suicide attempts, who were admitted to hospital within the same time frame as the cases. The community control group consisted of individuals with no history of suicide attempts who were recruited from community and non-psychiatric clinical areas. Participants were excluded if they were unable to provide informed consent or follow study procedures.

### Recruitment

Upon recruitment, fasting blood and urine samples were collected and a structured interview was conducted. Data were obtained on socio-demographic variables (age, sex, ethnicity, religion, marital status, education, employment, and social support), medical history and current medications, psychopathology, physical measurements, and suicidal behaviour. The study questionnaires were compiled using previously validated diagnostic and assessment tools including the Mini-International Neuropsychiatric Interview^38^ and the Beck Suicide Intent Scale^39^. For participants in the case group, a detailed description of the suicide attempt was recorded. All assessments were administered in hospital or community by trained research staff.

### Laboratory Analysis

12-hour fasting blood samples were collected, processed, and stored at the Clinical Research and Clinical Trials Laboratory at Hamilton General Hospital. Samples were processed within two hours of collection. After 30 minutes of clotting time, samples were spun at 1500 × g (3000 rpm) for 15 minutes until blood was well separated. Samples were then aliquotted into 2 mL cryovials and stored in liquid nitrogen for future analyses. Serum BDNF level was assayed using Quantikine^®^ ELISA Human BDNF Immunoassay (R&D Systems Inc.). All analyses were conducted blindly according to standard procedures.

### Statistical Analysis

We used descriptive statistics to summarize baseline characteristics of the sample. Means and standard deviations (SD) were reported for continuous variables, and counts and percentages were reported for categorical variables. Analysis of variance tests (ANOVA) were used to compare means for continuous variables and chi-square tests were used to compare proportions for categorical variables.

We employed a linear regression to model the association between attempted suicide and serum BDNF level. The confounders included in the model were selected a priori based on previous literature (age, sex, smoking status, body mass index, and antidepressant use). Serum BDNF level was the outcome variable, and case/control group (suicide attempt, psychiatric control, or community control) was the independent variable of interest. We chose to perform a linear regression analysis with BDNF level as the dependent variable because, while it is unknown whether BDNF is related to suicidal behaviour in a causal manner, it is known that many factors can influence BDNF levels. This analysis allowed us to investigate the relationship between attempted suicide and BDNF level in the context of other factors known to affect BDNF level.

We performed a sensitivity analysis using linear regression to explore the association between attempted suicide and serum BDNF level by comparing the case group to each control group individually. The same confounders listed above were included in the sensitivity analyses models. All analyses were performed using R version 3.0.2^40^.

## Additional Information

**How to cite this article**: Eisen, R.B. *et al*. Exploring the Association between Serum BDNF and Attempted Suicide. *Sci. Rep*. **6**, 25229; doi: 10.1038/srep25229 (2016).

## Figures and Tables

**Table 1:** Comparison of Baseline Characteristics.

	Cases (n = 84)	Psychiatric controls (n = 104)	Community controls (n = 93)	Univariate differences^1^
*Demographic Variables*				
Mean Age (SD) (years)	43.01 (14.03)	45.01 (14.23)	46.36 (17.81)	F = 1.05, P = 0.35
Sex (% female)	44 (52.38)	52 (50.00)	46 (49.46)	χ^2^ = 0.17, P = 0.92
Completed secondary education (%)	35 (42.68)	51 (50.49)	69 (74.19)	χ^2^ = 19.53, P < 0.001
Currently employed (%)	22 (26.51)	33 (32.67)	55 (59.14)	χ^2^ = 22.80, P < 0.001
Marital status				χ^2^ = 24.20, P < 0.001
Never married (%)	27 (32.53)	43 (41.75)	28 (30.11)	
Married/common law (%)	21 (25.30)	31 (30.10)	50 (53.76)	
Widowed/separated/divorced (%)	35 (42.17)	29 (28.16)	15 (16.13)	
*Prognostic Factors*				
Currently smoking (%)	35 (43.21)	35 (35.35)	11 (11.83)	χ^2^ = 22.84, P < 0.001
Taking antidepressants (%)	40 (47.61)	32 (30.77)	14 (15.05)	χ^2^ = 22.04, P < 0.001
Mean serum BDNF (SD) (ng/ml)	24.21 (7.19)	23.88 (6.95)	24.77 (7.19)	F = 0.297, P = 0.59
Mean body mass index (SD) (kg/m^2^)	27.88 (7.28)	29.78 (9.80)	27.73 (6.18)	F = 0.045, P = 0.83
*Psychiatric Diagnoses*^2^				
Major depressive disorder (%)	42 (63.63)	38 (41.30)	20 (21.74)	χ^2^ = 28.21, P < 0.001
Mood disorder (%)	63 (95.45)	76 (82.60)	26 (28.26)	χ^2^ = 95.22, P < 0.001
Anxiety disorder (%)	43 (65.15)	49 (53.26)	9 (9.78)	χ^2^ = 58.93, P < 0.001
Alcohol abuse (%)	18 (27.27)	13 (14.13)	4 (4.35)	P < 0.001
Substance abuse (%)	11 (16.67)	11 (11.96)	2 (2.17)	P = 0.003
Eating disorder (%)	5 (75.76)	5 (5.43)	1 (1.09)	P = 0.11
Psychotic disorder (%)	3 (4.55)	6 (6.52)	0 (0)	P = 0.024
Antisocial personality disorder (%)	15 (22.73)	0 (0)	2 (2.17)	P < 0.001

^1^Analysis of variance tests (ANOVA) were used to compare means for continuous variables. Chi-square tests were used to compare proportions for categorical variables. Fisher’s exact test was used for categorical variables when one or more values in the contingency table were below 5.

^2^Since not all participants underwent the Mini-International Neuropsychiatric Interview (MINI), the group sizes for this section of the table are as follows: 66 cases, 92 psychiatric controls, 92 community controls. Abbreviations: SD, standard deviation; BDNF, brain-derived neurotrophic factor.

**Table 2:** Association between BDNF and Key Baseline Characteristics.

	Univariate Analysis			Multivariable Analysis^1^		
Variables	β Estimate	Standard Error	P-value	β Estimate	Standard Error	P-value
Group						
Suicide attempt	−0.56	1.09	0.611	0.28	1.20	0.818
Psychiatric control	−0.89	1.04	0.391	−0.82	1.10	0.455
Community control	0 (ref.)	–	–	0 (ref.)	–	–
Age (years)	−0.02	0.03	0.388	−0.02	0.03	0.578
Sex (female)	1.29	0.87	0.138	1.51	0.89	0.090
Currently smoking (yes)	1.08	0.97	0.268	1.43	1.02	0.163
Body mass index (kg/m^2^)	−0.03	0.05	0.613	−0.01	0.05	0.864
Taking antidepressant (yes)	−2.06	0.92	0.026*	−2.50	0.99	0.012*

^1^Adjusted R-squared: 0.022. P-value: 0.09. N = 250.
